# Methacrylate: An alternative fixing agent for identifying the botanical origin of propolis

**DOI:** 10.1002/aps3.11309

**Published:** 2019-12-08

**Authors:** Érica W. Teixeira, Dejair Message, Renata M. S. A. Meira

**Affiliations:** ^1^ Honey Bee Health Specialized Laboratory Biological Institute São Paulo State Agribusiness Technology Agency Av. Prof. Manoel César Ribeiro 1920, Pindamonhangaba São Paulo 12411‐010 Brazil; ^2^ Department of Animal Science Federal Rural University of the Semiarid Km 47‐BR110, Mossoró Rio Grande do Norte 59625‐900 Brazil; ^3^ Department of Plant Biology Federal University of Viçosa Av. Peter Henry Rolfs s/n Viçosa Minas Gerais 36571‐000 Brazil

**Keywords:** *Apis mellifera*, botanical composition, historesin, plant fragment

## Abstract

**Premise:**

A new technique was developed to identify the botanical origin of propolis, a resin‐like material made by bees by mixing saliva and beeswax with plant buds and exudates, using methacrylate for permanent slide preparation.

**Methods and Results:**

Propolis samples were fixed in methacrylate to produce permanent slides. The anatomical structures of the plant fragments in the methacrylated propolis were compared with propolis slides prepared using conventional techniques that consist of propolis sediment obtained during a series of solvent reactions, filtration, and centrifugations, which cost a similar amount to produce. The techniques resulted in qualitative differences between the slides obtained. The methacrylated propolis sections allowed the detailed observation and identification of plant anatomical structures that were obscured in samples prepared using the conventional procedure. This clarity enabled the detailed evaluation of valuable taxon‐diagnostic characters in a permanent slide, which can also be used for histochemical tests.

**Conclusions:**

The methacrylated embedding of propolis is an affordable technique that could be implemented as a routine laboratory procedure. This new technique enables the efficient determination of the botanical origin of propolis.

Propolis is a bee product consisting mostly of beeswax and resin, which is collected by worker bees from plant buds or exudates (Salatino et al., 2019). A vast scientific literature has described various aspects of propolis, including the diversity of resin sources and the biological activities attributed to propolis extracts and isolated constituents (see Teixeira et al., 2003, 2005; Salatino et al., 2005; Toreti et al., 2013; Freitas et al., 2017; Bankova et al., 2018; Silva et al., 2019). Maciel et al. (2002) noted that the medicinal properties of propolis (i.e., anti‐inflammatory, antioxidant, and antimicrobial properties) have attracted the interest of researchers from varied fields of study (e.g., botany, pharmacology, phytochemistry), whose research has enriched our knowledge of natural medicinal sources.

Because propolis is mainly composed of compounds produced by plants, its chemical profile is derived from the contribution of the different plant species providing the resin (Bankova et al., 2018; Silva et al., 2019). Several factors influence the botanical composition of propolis, such as climatic conditions of the production area and the phenological variation of the vegetative species (Wink, 1990; Huang et al., 2014; Anjum et al., 2018). The confirmation that plant tissue fragments may be found in propolis samples allowed researchers to make use of known anatomical features to identify the plant species used in its production, which makes it possible to determine the plant source from which bees gathered exudate or buds (Oliveira and Bastos, 1998; Valcic et al., 1999; Freire, 2000; Montenegro et al., 2000, 2001; Bastos, 2001; Montenegro, 2001). The conventional methods typically used to identify the plant anatomical features in propolis are based on the procedure presented by Warakomska and Maciejewicz (1992), with a few modifications. Basically, the propolis sediment is obtained during a series of solvent reactions, filtrations, and centrifugations and spread onto slides for a visual inspection using microscopy. Using these techniques, the observation of the anatomical structures of plant fragments present in the samples was often difficult because the structures frequently overlapped; hence, the development of anatomical procedures aimed at obtaining higher‐quality images is necessary for the reliable diagnosis of the constituent plant fragments during propolis analyses.

Methacrylate, usually called historesin, is a useful alternative to conventional embedding media (e.g., histological paraffin). Methacrylate is a transparent synthetic resin embedding medium used for standard staining and sectioning with rotary microtomes. Over the past few decades, methacrylate has been frequently used in anatomical studies of both animal and plant tissues (Meira and Martins, 2003; Jesus Júnior et al., 2015) because of its high infiltration capacity, which enables the very thin sectioning of the desired sample, as well as the possibility of performing histochemical tests on the fixed tissues. The present work aimed to test the efficacy of using methacrylate in the preparation of propolis samples to facilitate the identification of the origin of plant fragments present.

## METHODS AND RESULTS

Propolis samples were collected monthly for 12 months (January to December 2001) from three experimental apiaries of an Africanized honey bee (*Apis mellifera* L.), each of which contained five colonies. The apiaries were located in Minas Gerais, Brazil, in three different municipalities: Itapecerica (20°32′S, 45°13′W), Paula Cândido (20°49′S, 42°54′W), and Virginópolis (18°50′S, 42°43′W). Langstroth‐style hives were used, with special brood chambers containing 3‐cm lateral slits to stimulate propolis production (see Salatino et al., 2005). After collection, the propolis samples were individually stored in glass vials at –20°C until required for analysis.

Compound samples (i.e., combined samples collected each month from the same location) were individually prepared from the propolis samples collected from the studied beehives. Each 2‐g propolis sample was transferred into a glass vial (total volume of 8 mL) containing 85% ethanol for 2 h. The supernatant was discarded, and the samples were placed on filter paper (InLab type 10, porosity 3 μm; InLab, Diadema, São Paulo, Brazil) to remove the excess solvent before being transferred to another glass vial containing 95% ethanol for 2 h. The supernatant was again discarded, and the samples were dried on filter paper before being transferred to new vials containing a 1 : 1 (v/v) mixture of 95% ethanol and infiltration resin solution. The infiltration resin solution contained 50 mL of liquid basic resin with 0.5 g of an activator powder, prepared according to the manufacturer's instructions (Leica HistoResin; Leica Biosystems, Wetzlar, Germany). Four hours later, the samples were filtered, and the residue retained on the paper was spread in Petri dishes and covered with filter paper (InLab type 10, porosity 3 μm) to remove the 95% ethanol–resin mixture. The resulting residue was submerged overnight in a glass vial containing infiltration resin, after which the sample was again spread on filter paper to dry. A spatula was used to transfer 0.4 g of the residue to the embedding resin solution prepared with a mix of 15 mL of the infiltration resin solution and 1 mL of hardener (Leica Biosystems), prepared according to the manufacturer's instructions. The product was then transferred into the 0.8 × 1.0‐cm wells of a plastic mold (HistoMold; Leica Biosystems). Each transfer, during either the ethanol series or the embedding process, was performed under a vacuum in a desiccator to achieve the proper infiltration.

The plastic molds containing the embedded samples were incubated in an oven at 34°C for 4 h, or until the polymerization of the resin was complete. The polymerized resin blocks containing the propolis samples were then removed from the molds, adhered to small wood supports, and stored in closed glass jars with silica until being sectioned.

The propolis sample blocks were sliced using a rotary microtome (Leica RM 2155; Leica Biosystems) with an automatic advance and a glass knife. Section thicknesses of 4, 8, 12, and 16 μm were tested, with the 12‐μm thickness providing the best quality for observing the morphological parameters of the plant fragments contained in the propolis samples. The sections were floated on distilled water and collected onto histological slides, as described by Leitão (2018). The slides containing the sections were dried on a hot plate to promote adhesion. Following O'Brien and McCully (1981), toluidine blue (pH 4) was used as the staining agent and was applied for an exposure time of 18 min at room temperature (20–23°C). After being dried on vertical supports at room temperature, the samples were mounted onto slides using synthetic resin (Permount; Thermo Fisher Scientific, Waltham, Massachusetts, USA). These slides were stored in wooden boxes as a permanent slide collection. The observational analysis and photographic documentation of the slides were carried out using a light microscope (AX70 TRF; Olympus, Shinjuku, Tokyo, Japan) equipped with a U‐Photo photographic system and a digital camera (Spot Insight Color 3.2.0; Diagnostic Instruments Inc., Sterling Heights, Michigan, USA).

The anatomical structures of the methacrylate‐embedded samples were compared with those visible on slides made using the procedures described by Warakomska and Maciejewicz (1992), an extraction method in which slides were prepared with propolis sediment obtained during a series of solvent reactions, filtration, and centrifugations. Both sample sets contained propolis collected at the same time and localities, as detailed above. The methodologies are referred to here as the “methacrylate embedding technique” and the “conventional technique.”

Branches of the plants near the beehives were collected, their taxonomic identities were confirmed, and the specimens were deposited in the Herbarium of the Department of Plant Biology, Federal University of Viçosa, Brazil (see Appendix 1). Plant species growing and collected near the apiaries were used a reference material for the anatomical structure comparisons between the slides prepared using the two methodologies, with a plant anatomy slide collection prepared using a standard technique (Johansen, 1940).

### Comparison of the methacrylate embedding technique and the conventional technique

The methacrylate inclusion technique enabled the detailed analysis of the plant tissues in the propolis samples, with none of the fragment overlap observed in the samples prepared using the conventional method. When the images obtained from both propolis slide collections were compared, marked qualitative differences were noted regarding the clarity of the visualization of the detected plant fragment anatomical structures.

**Figure 1:**
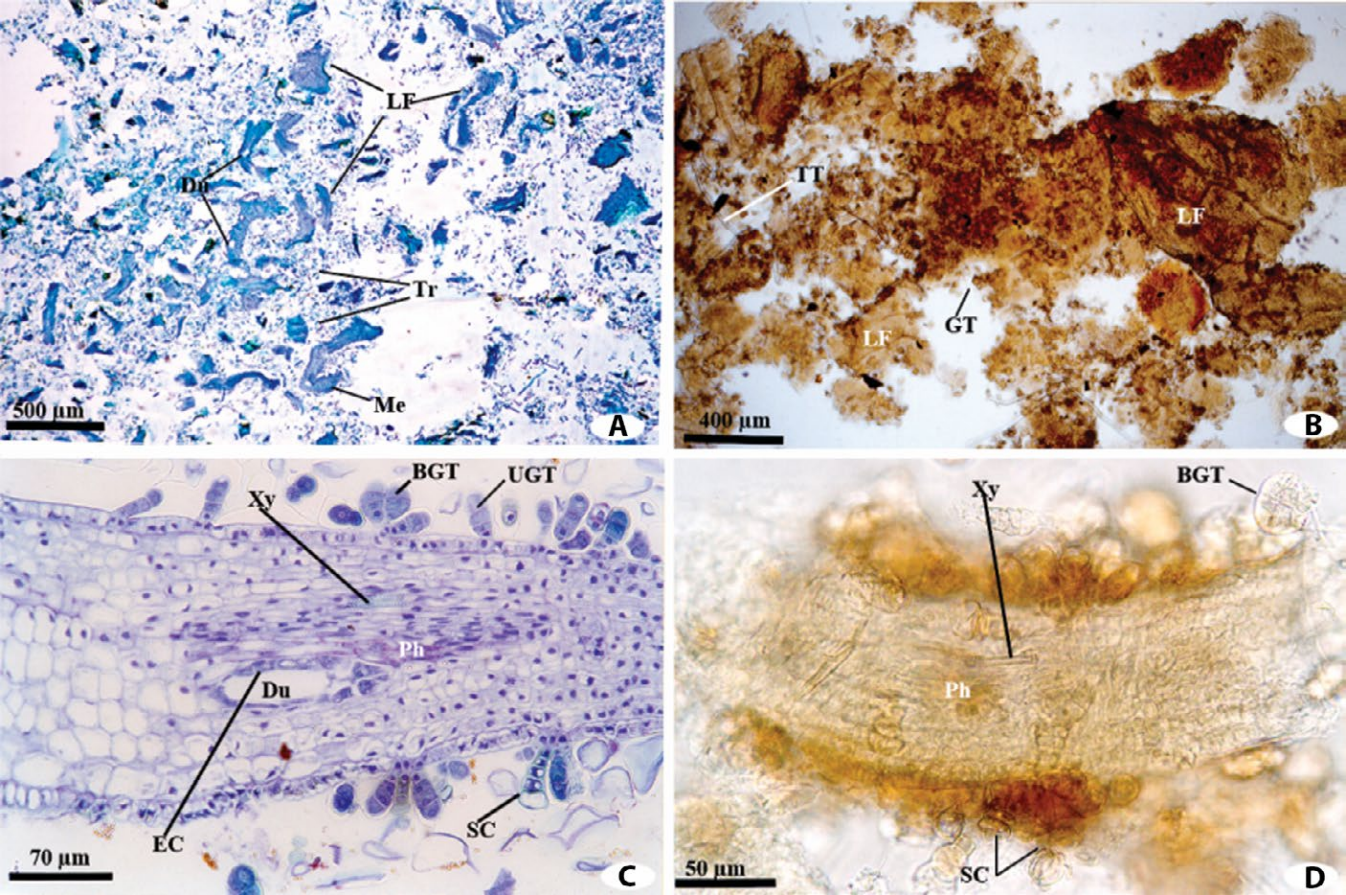
Overview of the anatomical structures observed in propolis from Paula Cândido embedded in methacrylate (A, C) or using the conventional technique (B, D). (A) Methacrylate‐embedded propolis containing fragments of *Baccharis dracunculifolia*. (B) Propolis sediment obtained using the conventional technique containing vegetative tissue fragments. (C) Detailed anatomical structures of the *B. dracunculifolia* leaf fragments observed in the propolis sample in Fig. 1A. (D) Detailed anatomical structures of the *B. dracunculifolia* leaf fragments observed in the propolis sample in Fig. 1B. BGT = biseriate glandular trichome; Du = duct; EC = epithelial cell; GT = glandular trichome; LF = leaf fragment; Me = mesophyll; Ph = phloem; SC = stalk cells; Tr = trichome; TT = tector trichome; UGT = uniseriate glandular trichome; Xy = xylem.

The differences between the two techniques can be demonstrated with *Baccharis dracunculifolia* DC. (Asteraceae), a common species found in close proximity to the hives, as an example. Fragments of *B. dracunculifolia* were abundant and predominant in the propolis samples from Itapecerica and Paula Cândido. The majority of the images used here to show the differences between the techniques applied for the propolis analysis depict fragments of *B. dracunculifolia*. An overview of the anatomical structures observed in the propolis samples from Paula Cândico and embedded either in methacrylate or using the conventional techniques are shown in Figures 1A and 1B. The methacrylate embedding technique enabled the identification of diagnostic internal features specific to *B. dracunculifolia*, such as resin ducts and their positions relative the vascular bundles, as well as the epidermal appendages (Fig. 1C). Using the conventional technique (Fig. 1D), such information was only obtained when the cuts made by the bee mandible in the mesophyll region were visualized, and even in these cases the image definition was poor.

A comparative analysis between the reference slide collection and the propolis slide collection prepared using the methacrylate technique highlighted the high degree of preservation in the samples collected by the bees. The anatomical structures of the *B. dracunculifolia* leaves were similar to those of the fragments found in the methacrylate‐embedded propolis samples (Fig. 1C); for example, uniseriate and biseriate secretory trichomes, tector trichomes, and ducts associated with vascular bundles turned toward the phloem were observed in the propolis. Such characteristics are consistent with previous leaf anatomy studies of *B. dracunculifolia* (Castro, 1987; Oliveira and Bastos, 1998; Freire, 2000; Bastos, 2001). The slide collection generated using the conventional technique did not allow the precise comparison of important details such as the mesophyll internal characters (Fig. 1D) because the fragments and structures contained in the slides overlapped, constraining the identification of the plant species.

**Figure 2:**
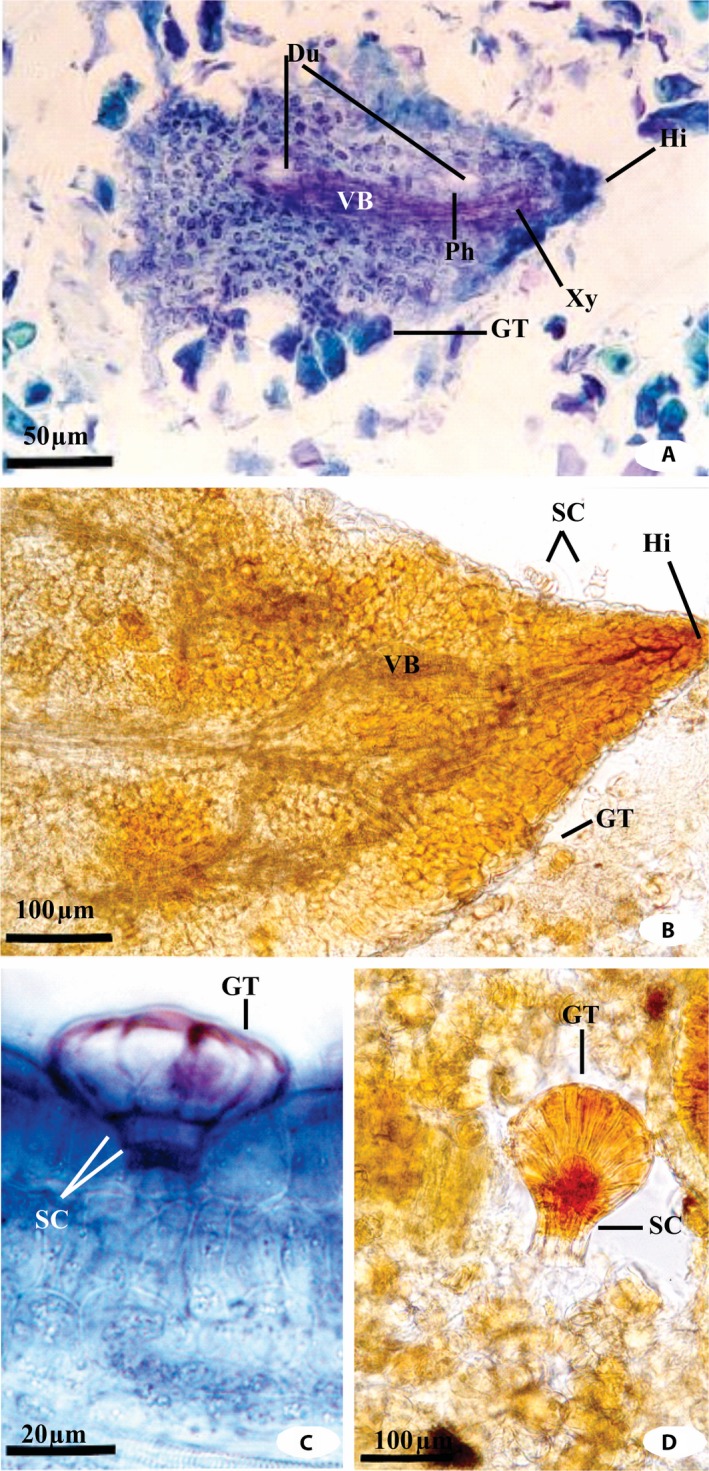
Specialized structures observed in propolis from Itapecerica and Paula Cândido embedded in methacrylate (A, C) or using the conventional technique (B, D). (A) Hydathodes from a *Baccharis dracunculifolia* leaf in the methacrylate‐embedded propolis from Itapecerica. (B) Hydathodes from a *B. dracunculifolia* leaf in a propolis sediment from Itapecerica prepared using the conventional technique. (C) Cross section of glandular trichome of *Myrsine umbellata* prepared using the methacrylate technique. (D) Glandular trichome of *M. umbellata* in a propolis sediment from Paula Cândido, prepared using the conventional technique. Du = duct; GT = glandular trichome; Hi = hydathode; Ph = phloem; SC = stalk cells; VB = vascular bundle; Xy = xylem.

Figures 1 and 2 show the specialized anatomical structures observed in propolis prepared with methacrylate or using the conventional technique. A few structures (e.g., the hydathodes) could be clearly visualized in the methacrylate‐embedded samples but could not be easily distinguished in those prepared using the conventional method (Fig. 2A). In propolis sediments obtained using the conventional technique, such structures were clearly visualized, although the organization of the vascular bundle was not recognizable (Fig. 2B). The secretory trichomes of *Myrsine umbellata* G. Don (Primulaceae) were easily identified in the samples prepared using the methacrylate technique (Fig. 2C). This led us to search for these structures in the propolis slide collection from Paula Cândido produced using the conventional technique, which ultimately allowed the detection of these fragments in these samples as well (Fig. 2D). In the propolis compound sample from Virginópolis, the tissues of plant sources other than *B. dracunculifolia* could be visualized using both techniques. The trichomes of *Vernonia polyanthes* (Spreng.) Less. (Asteraceae), tracheids of Gymnospermae, and free tector trichomes of *Sida* sp. (Malvaceae) were observed using both techniques, but the slides prepared in methacrylate provided clearer images with little to no overlap of the structures. These results highlight the utility of the methacrylate technique for the identification of the plant species contributing to the propolis. Considering the high plant diversity of the Brazilian biomes (Meira‐Neto and Martins, 2000, 2002; Silva et al., 2000, 2003), this approach could be extremely useful for identifying the botanical composition of propolis from different sources. This knowledge is important because the local flora influences both the diversity and the activities of propolis (Teixeira et al., 2003, 2005; Salatino et al., 2005; Toreti et al., 2013; Freitas et al., 2017; Bankova et al., 2018; Silva et al., 2019).

### Advantages of the methacrylate embedding technique

The methacrylate embedding technique enables the clearer visualization of all plant‐derived materials present in the propolis samples. The use of this technique reduces the loss of important diagnostic structures and organic components that can occur during the mass filtering and sieving required in the conventional technique (Warakomska and Maciejewicz, 1992; Barth, 1998). The methacrylate embedding technique also enables the visualization of the amorphous content (resulting from plant resin that is not associated with plant fragments) interspersed among the plant fragments and structures, which is not eliminated during the dehydration process using the ethanol series. This amorphous content likely originates from the leakage resulting from the breakdown of the internal and external plant secretory structures during the fragmentation of the foliar apices by the bees. It is feasible to produce a series of cross sections of a known thickness using this technique, which may be particularly useful for the comparison of anatomical structures because it prevents the overlap of fragments and provides clear images that allow the clear‐cut observation of important diagnostic characteristics.

A methacrylate kit (Leica HistoResin) allows for the preparation of around 2000 propolis samples, corresponding to a per‐unit cost of approximately US$0.60 per sample, while the conventional technique cost is approximately US$0.50 per sample. The advantages of methacrylate outweigh the small difference in price however; methacrylated propolis is more efficient because the slides have a longer durability (they may last over 50 years) and result in a permanent reference, as registered in the histology library (“Histothèque”) of the Muséum National d'Histoire Naturelle in Paris, France (https://www.mnhn.fr/en/collections/collection-groups/botany/histotheque-histology-library [accessed 4 November 2019]). In contrast, slide collections produced using the conventional technique are temporary, with a durability limited to only a few months. Moreover, the clearer samples produced using the methacrylate embedding method may facilitate the use of various histochemical tests, such as periodic acid–Schiff staining for the detection of total polysaccharides (McManus, 1948) and ruthenium red (Johansen, 1940) for the detection of mucilage and/or pectins.

## CONCLUSIONS

The low cost of preparation and the high image quality of propolis samples processed using the methacrylate embedding technique highlight the superiority of this approach over the conventional technique. The methacrylate technique enables the clearer visualization of all plant‐derived material originally present in the propolis sample, as well as the observation of the amorphous content interspersed among the plant fragments and structures. The generation of a series of cross sections of a known thickness (12 μm) may facilitate the use of various histochemical tests, and the slide collection can be used as a permanent reference. This technique could be implemented as a routine laboratory procedure for the analysis of propolis samples.

## APPENDIX 1

Species collected by Érica Weinstein Teixeira in three municipalities of Minas Gerais State, Brazil (vouchers deposited at the Herbarium of the Department of Plant Biology at the Federal University of Viçosa, Minas Gerais, Brazil [VIC]), and used in this study as a reference for identifying plant fragments in propolis samples.

**ITAPECERICA** (20°32′S, 45°13′W). Anacardiaceae: *Lythraea molleoides* (Vell.) Engl. (VIC 26822); *Mangifera indica* L. (VIC 26824). Asteraceae: *Ageratum conyzoides* L. (VIC 26889); *Ageratum fastigiatum* (Gardner) R. M. King & H. Rob. (VIC 26890, 26891, 26892, 26894, 26895); *Baccharis calvescens* DC. (VIC 26905, 26906); *Baccharis dracunculifolia* DC. (VIC 26915, 26916, 26921, 26922, 26923, 26924); *Bidens segetum* Mart. ex Colla (VIC 26882, 26881); *Eupatorium crenulatum* Gardner (VIC 26883); *Eupatorium maximiliani* Schrad. ex DC. (VIC 26884); *Eupatorium squalidum* DC. (VIC 26887); *Gochnatia polymorpha* (Less.) Cabrera (VIC 25231); *Vernonia polyanthes* (Spreng.) Less. (VIC 26171). Boraginaceae: *Cordia verbenacea* DC. (VIC 25538). Primulaceae: *Myrsine umbellata* Mart. (VIC 26801). **PAULA CÂNDIDO** (20°49′S, 42°54′W). Amarantaceae: *Alternanthera brasiliana* (L.) Kuntze (VIC 26820). Asteraceae: *Achyrocline satureioides* (Lam.) DC. (VIC 26879); *Baccharis dracunculifolia* DC. (VIC 25287, 26912, 26913, 26919, 26920); *Bidens pilosa* L. (VIC 25288); *Emilia sonchifolia* (L.) DC. ex Wight (VIC 25286); *Sonchus oleraceus* L. (VIC 25555); *Vernonia scorpioides* (Lam.) Pers. (VIC 25257, 26897). Bignoniaceae: *Pyrostegia venusta* (Ker Gawl.) Miers (VIC 26825, 26826). Leguminosae, Mimosoideae: *Stryphnodendron guianense* (Aubl.) Benth. (VIC 25560); *Piptadenia gonoacantha* (Mart.) J. F. Macbr. (VIC 25561). Oxalidaceae: *Averrhoa carambola* L. (VIC 25569). Rosaceae: *Eriobotrya japonica* (Spreng.) Lindl. (VIC 25570). Verbenaceae: *Aegiphila sellowiana* Cham. (VIC 25292); *Lantana camara* L. (VIC 12529). **VIRGINÓPOLIS** (18°50′S, 42°43′W). Asteraceae: *Ageratum fastigiatum* (VIC 26893); *Ageratum conyzoides* (VIC 26889); *Alomia fastigiata* Benth. (VIC 26878); *Baccharis calvescens* (VIC 26904, 26906); *Baccharis dracunculifolia* (VIC 26914, 26917, 26918); *Baccharis trimera* (Less.) DC. (VIC 26925); *Eupatorium squalidum* (VIC 26886, 26888); *Vernonia membranacea* Gardner (VIC 26896); *Vernonia polyanthes* (VIC 26900, 26901, 26902, 26903). Lauraceae: *Nectandra rigida* (Kunth) Nees (VIC 26833). Leguminosae, Papilionoideae: *Phaseolus vulgaris* L. (VIC 26836). Verbenaceae: *Lantana camara* (VIC 25264).

## References

[aps311309-bib-0001] Anjum, S. I. , A. Ullah , K. A. Khan , M. Attaullah , H. Khan , H. Ali , M. A. Bashir , et al. 2018 Composition and functional properties of propolis (bee glue): A review. Saudi Journal of Biological Sciences. 10.1016/j.sjbs.2018.08.013. PMC6864204 31762646

[aps311309-bib-0002] Bankova, V. , M. Popova , and B. Trusheva . 2018 The phytochemistry of the honeybee. Phytochemistry 155: 1–11. 30053651 10.1016/j.phytochem.2018.07.007

[aps311309-bib-0003] Barth, O. M. 1998 Pollen analysis of Brazilian propolis. Grana 37: 97–101.

[aps311309-bib-0004] Bastos, E. M. A. F. 2001 Origem botânica e indicadores de qualidade da “própolis verde” produzida no Estado de Minas Gerais. Ph.D. thesis, Faculdade de Filosofia, Ciências e Letras, Ribeirão Preto, Universidade de São Paulo, São Paulo, Brazil Website http://bdpi.usp.br/single.php?_xml:id=001211801 [accessed 11 November 2019] (in Portuguese).

[aps311309-bib-0005] Castro, M. M. 1987 Estruturas secretoras em folhas de espécies da família Asteraceae: aspectos estruturais e histoquímicos. Ph.D. thesis, Instituto de Biociências, Universidade de São Paulo, São Paulo, Brazil (in Portuguese).

[aps311309-bib-0006] Freire, U. C. 2000 Origem da própolis verde e preta produzida em Minas Gerais. M.S. dissertation, Universidade Federal de Viçosa, Viçosa, Brazil Website http://www.locus.ufv.br/handle/123456789/9729?show=full [accessed 11 November 2019] (in Portuguese with English abstract).

[aps311309-bib-0007] Freitas, M. C. D. , M. B. de Miranda , D. T. de Oliveira , S. A. Vieira‐Filho , R. B. Caligiorne , and S. M. de Figueiredo . 2017 Biological activities of red propolis: A review. Recent Patents on Endocrine, Metabolic & Immune Drug Discovery 11: 3–12. 10.2174/1872214812666180223120316 29473533

[aps311309-bib-0008] Huang, S. , C.‐P. Zhang , K. Wang , G. Q. Li , and F.‐L. Hu . 2014 Recent advances in the chemical composition of propolis. Molecules 19: 19610–19632. 25432012 10.3390/molecules191219610 PMC6271758

[aps311309-bib-0009] Jesus Júnior, L. A. , R. P. Oliveira , and K. R. B. Leite . 2015 Improving microtechniques for processing leaf blades of grasses using *Ichnanthus pallens* (Sw.) Munro ex Benth. as a model species. Neodiversity 8: 50–54.

[aps311309-bib-0010] Johansen, D. A. 1940 Plant microtechnique. McGraw‐Hill Book Co. Inc., New York, New York, USA.

[aps311309-bib-0011] Leitão, C. A. E. 2018 Working optimally with serial sections in glycol methacrylate resin. Brazilian Archives of Biology and Technology 61: e18180103.

[aps311309-bib-0012] Maciel, M. A. M. , A. C. Pinto , and V. F. Veiga Jr . 2002 Plantas medicinais: A necessidade de estudos multidisciplinares. Química Nova 25: 429–438 (in Portuguese with English abstract).

[aps311309-bib-0013] McManus, J. F. A. 1948 Histological and histochemical uses of periodic acid. Stain Technology 23: 99–108. 18867618 10.3109/10520294809106232

[aps311309-bib-0014] Meira, R. M. S. A. , and F. M. Martins . 2003 Inclusão de material herborizado em metacrilato para estudos de anatomia vegetal [The inclusion of herbalized material using methacrylate for plant anatomy studies]. Revista Árvore 27: 109–112 (in Portuguese with English abstract).

[aps311309-bib-0015] Meira‐Neto, J. A. A. , and F. R. Martins . 2000 Composição florística do estrato herbáceo‐arbustivo de uma floresta estacional semidecidual em Viçosa‐MG. Revista Árvore 24: 407–416 (in Portuguese).

[aps311309-bib-0016] Meira‐Neto, J. A. A. , and F. R. Martins . 2002 Composição florística de uma floresta estacional semidecidual montana no município de Viçosa‐MG. Revista Árvore 26: 437–446 (in Portuguese).

[aps311309-bib-0017] Montenegro, G. 2001 Botanical origin and seasonal production of propolis in hive of Central Chile. Boletim de Botânica da Universidade de São Paulo 19: 1–6.

[aps311309-bib-0018] Montenegro, G. , B. N. Timmermann , P. C. Pena , A. M. Mujica , and G. Avila . 2000 Pollen grains and vegetative structures in propolis as indicators of potential drugs in Chilean plants. Phyton 66: 15–23.

[aps311309-bib-0019] Montenegro, G. , A. M. Mujica , and R. Pizarro . 2001 Botanical resources for propolis in an apiary network in Central Chile. Phyton 50: 191–201.

[aps311309-bib-0020] O'Brien, T. P. , and M. E. McCully . 1981 The study of plant structure principles and selected methods. Termarcarphi Pty. Ltd., Melbourne, Australia.

[aps311309-bib-0021] Oliveira, V. C. , and E. M. Bastos . 1998 Aspectos morfo‐anatômicos da folha de *Baccharis dracunculifolia* DC. (Asteraceae) visando à identificação da origem botânica da própolis. Acta Botanica Brasilica 12(Suppl): 431–439 (in Portuguese with English abstract).

[aps311309-bib-0022] Salatino, A. , É. W. Teixeira , N. Giuseppina , and D. Message . 2005 Origin and chemical variation of Brazilian propolis. Evidence‐Based Complementary and Alternative Medicine 2: 33–38. 15841276 10.1093/ecam/neh060 PMC1062153

[aps311309-bib-0023] Salatino, A. , L. R. L. Pereira , and M. L. F. Salatino . 2019 The emerging market of propolis of stingless bees in tropical countries. MOJ Food Processing and Technology 7: 27–29.

[aps311309-bib-0024] Silva, A. F. , N. R. L. Fontes , and H. F. Leitão‐Filho . 2000 Composição florística e estrutura horizontal do estrato arbóreo de um trecho da Mata da Biologia da Universidade Federal de Viçosa‐Zona da Mata de Minas Gerais. Revista Árvore 24: 397–405 (in Portuguese).

[aps311309-bib-0025] Silva, A. F. , R. V. Oliveira , N. R. L. Santos , and A. de Paula . 2003 Composição florística e grupos ecológicos das espécies de um trecho de floresta semidecídua submontana da Fazenda São Geraldo, Viçosa‐MG. Revista Árvore 27: 311–319 (in Portuguese).

[aps311309-bib-0026] Silva, C. C. F. , A. Salatino , L. B. Motta , G. Negri , and M. L. F. Salatino . 2019 Chemical characterization, antioxidant and anti‐HIV activities of a Brazilian propolis from Ceará state. Revista Brasileira de Farmacognosia 29: 309–318.

[aps311309-bib-0027] Teixeira, E. W. , D. Message , R. M. S. A. Meira , and A. Salatino . 2003 Indicadores da origem botânica da própolis: Importância e perspectivas. Boletim da Indústria Animal 60: 83–106 (in Portuguese).

[aps311309-bib-0028] Teixeira, E. W. , N. Giuseppina , R. M. S. A. Meira , D. Message , and A. Salatino . 2005 Plant origin of green própolis: Bee behavior, plant anatomy and chemistry. Evidence‐Based Complementary and Alternative Medicine 2: 85–92. 15841282 10.1093/ecam/neh055 PMC1062148

[aps311309-bib-0029] Toreti, V. C. , H. H. Sato , G. M. Pastore , and Y. K. Park . 2013 Recent progress of propolis for its biological and chemical compositions and its botanical origin. Evidence‐Based Complementary and Alternative Medicine 2013: 697390. 23737843 10.1155/2013/697390 PMC3657397

[aps311309-bib-0030] Valcic, S. , G. Montenegro , A. M. Mujica , G. Avila , S. Franzblau , M. P. Singh , W. M. Maiese , and B. N. Timmermann . 1999 Phytochemical, morphological, and biological investigations of propolis from Central Chile. Zeitschrift für Naturforschung C 54: 406–416. 10.1515/znc-1999-5-617 10431392

[aps311309-bib-0031] Warakomska, Z. , and W. Maciejewicz . 1992 Microscopic analysis of propolis from Polish regions. Apidologie 23: 277–283.

[aps311309-bib-0032] Wink, M. 1990 Physiology of secondary product formation in plants *In* Charlwood B. V. and Rhodes M. J. C. [eds.], Secondary products from plant tissue culture, 67–86. Clarendon Press, Oxford, United Kingdom.

